# Empathy dimensions and their association with Borderline Personality Disorder symptomatology: a protocol for a retrospective study

**DOI:** 10.1192/j.eurpsy.2026.10966

**Published:** 2026-07-31

**Authors:** P. de Moura, M. Queiroz, V. Gonçalves, E. Osório, A. R. Ferreira, R. Barranha

**Affiliations:** 1 Department of Clinical Neurosciences and Mental Health, University of Porto, Faculty of Medicine; 2 Department of Psychology; 3 Department of Psychiatry, Unidade de Saúde Local de São João; 4 RISE-Health , Porto, Portugal

## Abstract

**Introduction:**

Borderline Personality Disorder (BPD) is a severe and complex mental condition characterized by emotional instability, impulsivity, and interpersonal difficulties. Empathy, encompassing both its cognitive and affective dimensions, is a core component of mentalizing capacity and constitutes one of the four domains outlined in the DSM-5-TR alternative model for personality disorders. The influence of empathy on interpersonal functioning of individuals with BPD underscores the importance of investigating how its different dimensions relate to specific BPD symptoms and overall symptoms severity.

**Objectives:**

To investigate the associations between distinct empathy dimensions and the severity and specific manifestations of BPD symptomatology.

**Methods:**

This retrospective study will include adult patients with a clinical diagnosis of BPD who have been followed at the psychiatry service of a university hospital. Those who have completed both the Borderline Symptom List (BSL) and the Interpersonal Reactivity Index (IRI) will be selected for analysis. Descriptive statistics will be used to characterize the sample, and Spearman’s correlation coefficient will be applied to explore associations between empathy dimensions and symptom domains. The study has received authorization from the hospital’s Ethics Committee (Aut. n.° 337-23).

**Results:**

A total of 106 adults comply with the study criteria. The results are expected to shed light on the hypothesized association between overall symptom severity and empathy dimensions, as well as whether specific symptoms may exhibit clinically meaningful associations with distinct empathy subscales.

**Conclusions:**

This methodological approach will allow a detailed examination of how empathy dimensions relate to BPD symptomatology. Such findings are anticipated to contribute to a better understanding of interpersonal functioning in BPD and inform the development of psychotherapeutic strategies tailored to individual empathy profiles.

**Disclosure of Interest:**

None Declared

